# Elevated Levels of Procoagulant Plasma Microvesicles in Dialysis Patients

**DOI:** 10.1371/journal.pone.0072663

**Published:** 2013-08-02

**Authors:** James O. Burton, Hassan A. Hamali, Ruchir Singh, Nima Abbasian, Ruth Parsons, Amit K. Patel, Alison H. Goodall, Nigel J. Brunskill

**Affiliations:** 1 Department of Infection, Immunity and Inflammation, University of Leicester, Leicester, United Kingdom; 2 Department of Renal Medicine, Leicester General Hospital, Leicester, United Kingdom; 3 Department of Cardiovascular Sciences, University of Leicester, Leicester, United Kingdom; 4 Department of Clinical Laboratory Sciences, King Khalid University, Abha, Saudi Arabia; 5 Department of Paediatric Emergency Medicine, Maimonides Medical Center, New York, United States of America; 6 Leicester National Institute for Health Research Biomedical Research Unit in Cardiovascular Disease, Glenfield Hospital, Leicester, United Kingdom; University of Sao Paulo Medical School, Brazil

## Abstract

Cardiovascular (CV) death remains the largest cause of mortality in dialysis patients, unexplained by traditional risk factors. Endothelial microvesicles (EMVs) are elevated in patients with traditional CV risk factors and acute coronary syndromes while platelet MVs (PMVs) are associated with atherosclerotic disease states. This study compared relative concentrations of circulating MVs from endothelial cells and platelets in two groups of dialysis patients and matched controls and investigated their relative thromboembolic risk. MVs were isolated from the blood of 20 haemodialysis (HD), 17 peritoneal dialysis (PD) patients and 20 matched controls. Relative concentrations of EMVs (CD144^+ ve^) and PMVs (CD42b^+ ve^) were measured by Western blotting and total MV concentrations were measured using nanoparticle-tracking analysis. The ability to support thrombin generation was measured by reconstituting the MVs in normal plasma, using the Continuous Automated Thrombogram assay triggered with 1µM tissue factor. The total concentration of MVs as well as the measured sub-types was higher in both patient groups compared to controls (p<0.05). MVs from HD and PD patients were able to generate more thrombin than the controls, with higher peak thrombin, and endogenous thrombin potential levels (p<0.02). However there were no differences in either the relative quantity or activity of MVs between the two patient groups (p>0.3). Dialysis patients have higher levels of circulating procoagulant MVs than healthy controls. This may represent a novel and potentially modifiable mediator or predictor of occlusive cardiovascular events in these patients.

## Introduction

Cardiovascular disease remains one of the largest causes of mortality and morbidity in patients with chronic kidney disease (CKD) and on renal replacement therapy [1]. However, the increased incidence and prevalence of cardiovascular disease in CKD and advancing uraemia cannot be accounted for using traditional models and risk profiles for the development of atherosclerosis in the general population [2].

One potential mechanism by which cardiovascular risk may be increased in uraemic patients may relate to dysfunctional haemostasis. The uraemic milieu causes defects in platelet function manifest as diminished adherence to the vascular sub-endothelium [3] through decreased expression of glycoprotein (GP) Ib [4] and dysfunction of GPIIb-IIIa, at least partly caused by fibrinogen fragments in the circulation that bind to and block the fibrinogen binding site [5]. Although circulating levels of fibrinogen and von Willebrand factor are normal [6], levels of coagulation factors are perturbed in end stage renal patients undergoing dialysis [7], all of which might be expected to contribute to a bleeding diathesis.

However there is good evidence that end-stage renal disease patients experience a high incidence of thrombotic events, including arteriovenous access thrombosis in patients on haemodialysis [8], spontaneous deep vein thrombosis, and pulmonary embolism [9,10], highlighting that acute vascular occlusion can occur in the presence of uraemia. A number of potential mechanisms may contribute to this pro-thrombotic tendency including platelet hyperaggregability [11–13] and the presence of circulating procoagulant microvesicles (MVs) [14,15]

Microvesicles are membrane bound particles shed from various parent cells, upon activation or apoptosis in response to various stimuli [16]. Within the blood microvesicles principally originate from endothelial cells, platelets and monocytes. Both endothelial and platelet derived MVs have been shown to have pro-coagulant properties which may be 50- to 100-fold higher than that of activated platelets [17,18]. The procoagulant activity is due to exposure of a negatively charged phospholipid surface that permits the assembly of the procoagulant tenase and prothrombinase complexes. In addition, MVs have been shown to promote the ability of tissue factor (TF) to initiate coagulation and facilitate the formation of coagulation complexes [19] as well as to directly initiate platelet agglutination [20]. Consequently MVs have been associated with acute coronary syndromes, high risk coronary lesions, strokes and peripheral arterial disease [21–24], which together are the predominant contributors to the cardiovascular mortality and morbidity observed in dialysis patients. Elevated levels of EMVs independently correlate with subclinical atherosclerosis and poor cardiovascular outcomes in patients with end-stage renal disease [25,26]. Interestingly, standard therapies already used to reduce cardiovascular risk may also have a role in modulating the cardiovascular effects of MVs. Losartan and simvastatin have been shown to decrease levels of PMVs in hypertensive, hyperlipidaemic and diabetic patients [27]. In addition, statins have also been shown to inhibit platelet deposition and aggregation as well as suppressing expression of tissue factor [28].

Although there is evidence that MVs in dialysis patients may express pro-coagulant characteristics [29], no studies to date have been undertaken to investigate the pro-thrombotic effect of MVs in the plasma of dialysis patients. We hypothesised that elevated levels of endothelial and platelet derived MVs may directly contribute to thrombogenesis in uraemic patients on dialysis, and that this may be a potential mechanism for increased risk of acute occlusive cardiovascular events in this group.

## Methods

### Ethics statement

Ethics approval for this study was granted by the Local Research Ethics Committee for Leicestershire, Northamptonshire and Rutland (LREC 05/Q2502/80). Written informed consent was obtained from all participants.

### Patients and controls

Twenty haemodialysis (HD) patients, 17 peritoneal dialysis (PD) patients and 20 healthy controls (HC) were studied. Dialysis patients were recruited from the prevalent HD and PD programmes at the Leicester General Hospital. Exclusion criteria included: on dialysis for less than four months; access via a tunnelled venous catheter or arterio-venous graft (HD patients); presence or suspicion of an intercurrent infection or known blood-borne virus and; inability to give informed consent. All HD patients therefore had native arterio-venous fistulae (AVF) and dialysed for 3.5 hours three times per week. The majority of the dialysis patients were treated with individualised doses of recombinant human erythropoietin, which was administered intravenously three times a week in the HD patients and subcutaneously once weekly in the PD patients.

The control population was recruited from healthy subjects at the University of Leicester, with no reported chronic kidney disease. Each group was matched for age, gender and reported cardiovascular co-morbidities.

### Blood collection and microvesicle isolation

In all subjects blood samples were drawn into 3mL S-monovette^®^ vacutainers (Sarstedt AG, Nümbrecht, Germany) containing 0.43mL of 0.106M trisodium citrate anticoagulant. HD patients’ blood samples were drawn before the start of dialysis prior to the administration of any anticoagulants. PD patients and healthy subjects were bled using a loosely fastened tourniquet and 21-gauge needle. Platelet free plasma (PFP) was removed from samples that were prepared using two centrifugation steps of 1,500 x *g* for 15 minutes followed by 13,000 x *g* for 2 minutes and then stored in 250µL aliquots at -80° C until subsequent use.

After thawing, MV pellets were prepared using established methods to optimise yield [30]. Briefly, 250µL of PFP was centrifuged at 18,000 x *g* for 30 minutes after which the top 225µL (90%) of plasma was removed and replaced by phosphate-buffered saline (PBS; 10mM phosphate buffer, 2.7mM KCl, 137mM NaCl, pH 7.2). The MVs were then resuspended and re-pelleted ready for further analysis.

### Electron microscopy

Qualitative assessment of the microvesicles was made by electron microscopy. Microvesicles in prepared pellets were visualised by transmission electron microscopy (TEM) (JEOL 1220, Tokyo, Japan) using both negative staining and after embedding in 3% agar and *Spurr*’s resin. Digital images were recorded using SIS Megaview III digital camera with analySIS software.

### Immunoblotting

Analyses were performed in duplicate by two separate individuals. For each subject in each group, identical volumes of PFP and reagents were used to pellet, solubilise and load MVs for analysis, allowing comparison of relative MV concentrations between subjects on the same gel. The optical density of the bands of interest were also compared to identical volumes of a standard preparation of either cultured human umbilical vein endothelial cells (HUVECs) or blood-derived platelets that had been previously prepared and stored in aliquots using the same methods. This allowed for comparison to be made between samples on different gels. MV pellets were solubilised on ice for 30 minutes with occasional vortexing using 75µL NP-40 buffer (49.5mM Tris, pH 8; 150mM NaCl; 1% Nonidet P-40; 1% phenylmethylsulfonyl fluoride) and subsequently an additional 100µL Laemmli 2x buffer (22.3mM Tris, pH 6.8; 20% glycerol; 4% sodium dodecylsulphate; 0.0025% bromophenol blue and 100mM dithiothreitol). Each gel was loaded with a molecular weight marker, the lysate controls and preparations from two subjects from each of the control, HD and PD groups. Membrane proteins were then separated by SDS-PAGE in 8% SDS gels and transferred onto 0.2mm nitrocellulose membranes before blocking with 20% milk protein in PBS containing 0.1% Tween-20 (PBST). Membranes were incubated overnight at 4° C in PBST with 1:1000 dilutions of either anti-CD144 (AbD Serotec, Kidlington, UK) or anti-CD42b antibodies (RFGP37; in house MAb [31]) as markers of endothelial and platelet MVs respectively. Finally, membranes were incubated for one hour at room temperature in PBST with peroxidise-conjugated antibodies (Sigma-Aldrich, Gillingham, UK) at a concentration of 1:40,000. Immunoreactive bands were visualized using an enhanced chemiluminescence detection system (Amersham ECL reagents, GE Healthcare, Bucks, UK) and exposure to photographic film. The optical density of relevant bands was measured using Scion Image for Windows Alpha 4.0.3.2 (Scion Corporation, USA). Each sample’s optical density reading was then divided by the optical density of the standardised control to give a relative quantification of microvesicle concentration between patient and control groups.

### Nanoparticle tracking analysis

Absolute numbers and the size range of MVs measuring between 0.1–1µm were enumerated using nanoparticle tracking analysis (NTA) (NanoSight LM10 and NTA Software v 2.2, NanoSight Ltd, Amesbury, UK). Before measurement of each sample, the NTA apparatus was cleaned and flushed with twice-filtered 70% ethanol followed by ultra filtered nanopure water. MV samples were prepared from pellets as above and a background vesicle count was measured in filtered buffers. Patient samples were then analysed in duplicate using techniques detailed elsewhere [32] but with an enhanced 90-second video capture to increase the reproducibility of measurements before adjusting for background buffer debris.

### Measurement of thrombin generation using Calibrated Automated Thrombography

MVs were reconstituted in normal pooled plasma and thrombin generation was measured in samples of PPP from the patients and controls. This analysis assesses several main parameters of which three have been included for this study; the lag time (LT) is the length of time between the start of the reaction until the thrombin burst occurs; peak thrombin (PT) is the maximum amount of thrombin generated, and the endogenous thrombin potential (ETP) represents the total amount of thrombin generated over the course of the reaction and is calculated from the area under the curve. Each of these variables was measured in the plasma using Calibrated Automated Thrombography (CAT), essentially as previously described [33] using the PRP reagent comprising 1 pM tissue factor (Diagnostica Stago, Reading, UK), which is sensitive to the contribution of the phospholipid component of the MVs. Twenty microlitres of the PRP reagent were added to 80µL of plasma in Immulon 2HB round-bottomed microtitre plates and samples were incubated for 60 min at 37° C with continuous monitoring in a fluorescent plate reader equipped with Thrombinoscope software (Thrombinoscope, Synapse BV, Maastricht, Netherlands).

The PPP samples were analysed in parallel with paired samples that were filtered through a 0.22µM filter using a 96 well manifold vacuum filtration device (Ceveron®MFU 500 filtration unit: Technoclone, Dorking, UK). This removes MVs and thus acts as a control for other uraemic plasma factors that may affect thrombin generation.

As an additional control, for two samples from each subject group the MVs were isolated (using the same methods as described above), and reconstituted with filtered plasma to confirm that the observed differences were due to filtration of MVs and not other plasma products.

### Statistical analysis

Results are presented as the mean±standard deviation or the median and interquartile range unless otherwise stated. Gaussian distribution was assessed using the D’Agostino & Pearson normality test. Categorical variables between groups were analysed using the Chi squared test. The *t*-test and Mann-Whitney test for independent variables were used depending on results of the normality tests. Differences in size distribution were tested using the one-way analysis of variance (ANOVA). Correlations between groups of data were tested using Pearson’s test. All data were analysed using Prism v6 (GraphPad Software, La Jolla, CA, USA) and p<0.05 reflects statistical significance.

## Results

Baseline characteristics of patients and healthy subjects are given in table 1. There were no significant differences between the groups with respect to age, gender, smoking status or reported cardiovascular co-morbidities. The incidence of diabetes was significantly higher in the haemodialysis patients in line with the higher prevalence in this patient group.

**Table 1:** Demographics for each of the subject groups.

	HD Group (n=20)	PD Group (n=17)	Control Group (n=20)	p value
Age (years)	54.2±8.4	52.9±11.8	52.6±7.9	0.84
Male gender (n)	10	6	10	0.59
Hypertension (n)	5	4	2	0.42
IHD (n)	7	2	2	0.09
CVD (n)	3	1	0	0.17
PVD (n)	1	1	0	0.57
Hypercholesterolaemia (n)	3	4	2	0.53
Diabetes (n)	10	2	1	<0.01
Smoking status (n)	0	1	1	0.57
On statin therapy (n)	13	10	2	<0.001
On ACEi / ARB therapy (n)	11	11	1	<0.001

Data are shown as mean±SD (for age) or as the number of subjects in each category. p-values were calculated by t-test for the continuous variable of age or by Chi-squared analysis. HD, haemodialysis; PD, peritoneal dialysis; IHD, ischaemic heart disease; CVD, cerebrovascular disease; PVD, peripheral vascular disease; ACEi, angiotensin converting enzyme inhibitor and; ARB, angiotensin receptor blocker.

### Presence of MVs in plasma samples of patients and controls

**Figure 1:**
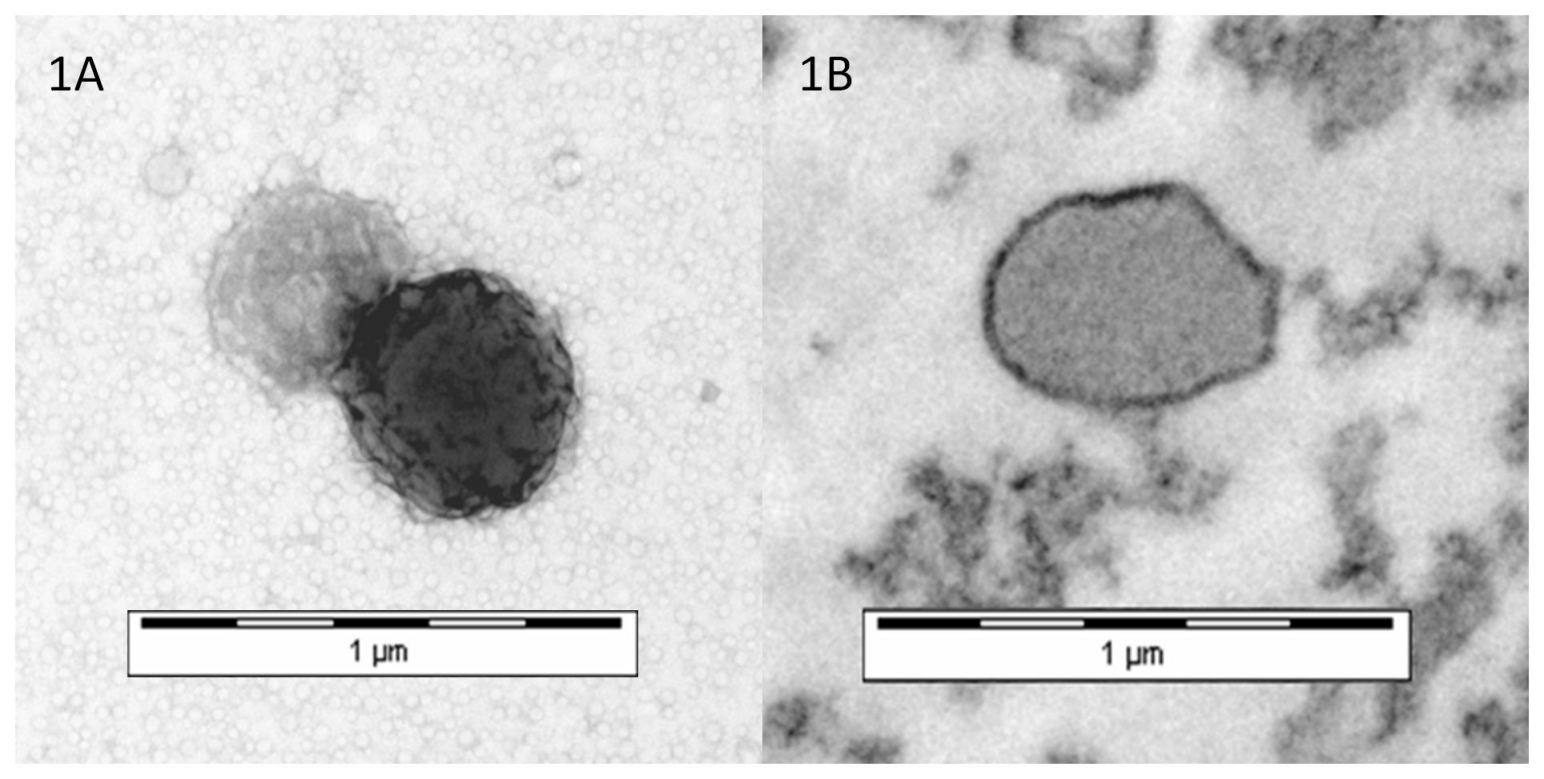
Example electron micrographs demonstrating the presence of microvesicles (MVs) prepared from normal plasma. (A) Negatively stained transmission electron micrograph showing two MVs of approximately 400nm and 500nm and (B) an embedded and sectioned transmission electron micrograph of an MV showing a bilayer appearance lacking any intracellular structures.

Electron microscopy in both patient and control groups confirmed the presence of distinct membrane bound vesicles of less than 1µm with a typical bi-layer appearance and no intracellular structures (Figure 1), consistent with current definitions of cell derived microvesicles [34].

**Figure 2:**
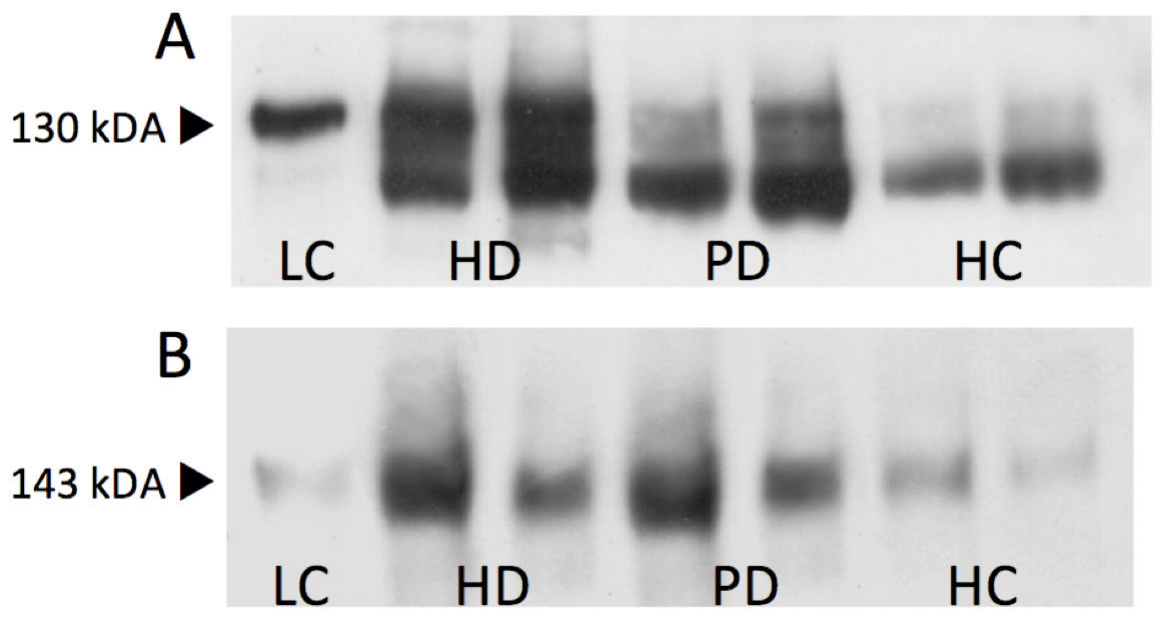
Representative Western blot results demonstrating the presence of both CD144+ ve endothelial (A) and CD42b+ ve platelet (B) derived MVs. LC, lysate control; HD, haemodialysis; PD, peritoneal dialysis; HC, healthy control.

Western blot analysis demonstrated the presence of both endothelial and platelet derived MVs by identifying specific membrane proteins (CD144 and CD42b respectively) within the MV preparations (Figure 2). Note the presence of a cleavage product (~104kDa) present in the patients’ samples immunoblotted with the CD144 antibody [35].

### Circulating levels of PMVs and EMVs are higher in patients on dialysis than controls

**Figure 3:**
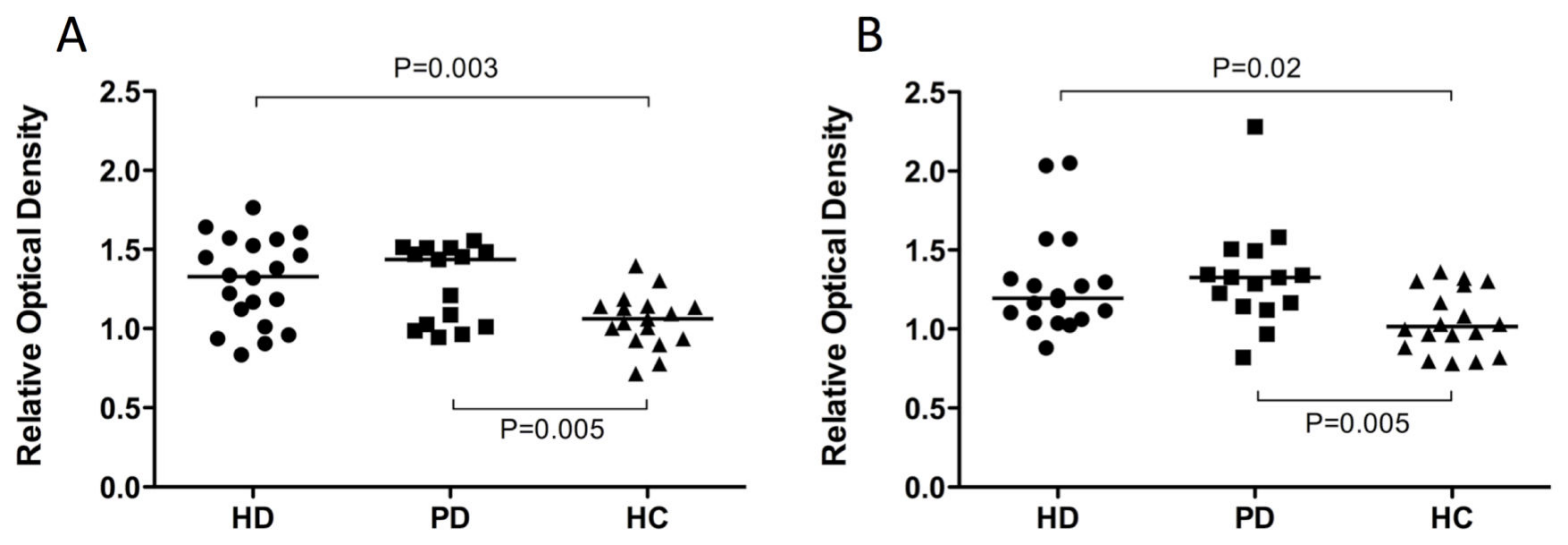
Relative concentrations of endothelial microvesicles (A) and platelet microvesicles (B) in dialysis patients compared to healthy controls. Graphs show relative optical density measurements against a standardised endothelial or platelet lysate control in each of the three subject groups. Horizontal bars represent the mean (A) or median (B). HD, haemodialysis; PD, peritoneal dialysis; HC, healthy control.

The relative concentration of EMVs was significantly higher in dialysis patients compared to healthy controls (1.29±0.26 vs. 1.05±0.17; p=0.003). When sub-divided, both the patients on haemodialysis, and those on peritoneal dialysis, had significantly higher concentrations of EMVs than controls (1.3±0.27 vs. 1.05±0.17; p=0.003 and 1.28±0.25 vs. 1.05±0.17; p=0.005 respectively, Figure 3A), but there was no difference in concentrations of EMVs between the two dialysis modalities (p=0.85).

Results for PMVs were similar (Figure 3B), with relative concentrations of PMVs significantly higher in dialysis patients compared to the controls (1.27, IQR 1.11-1.42 vs. 0.99, IQR 0.84-1.3). As with the EMVs, patients on haemodialysis and those on peritoneal dialysis each had higher concentrations of PMVs than controls (1.19, IQR 1.06-1.38 vs. 1.02, IQR 0.87-1.29; p=0.02 for the HD group; 1.33, IQR 1.14-1.49 vs. 1.02, IQR 0.87-1.29; p=0.005 for the PD patients), and there was no difference in concentrations of PMVs between the two dialysis modalities (p=0.3).

In addition there was a significant positive correlation in the levels of EMVs and PMVs in all dialysis patients, (*r* = 0.37, p=0.01).

### Total circulating levels of MVs are higher in dialysis patients vs. controls

To confirm the relatively higher levels of MVs in the patient groups the mean number of circulating MVs was measured using nanoparticle-tracking analysis. This analysis was carried out in only 21/57 samples (5 control samples, 7 haemodialysis and 9 peritoneal dialysis samples) due to limitations in the amount of plasma available. The total MV count was significantly higher in both haemodialysis and peritoneal dialysis patients compared to controls (76±33 x10^6^/mL and 33±18 vs. 19±2 x10^6^/mL respectively; p<0.05).

**Figure 4:**
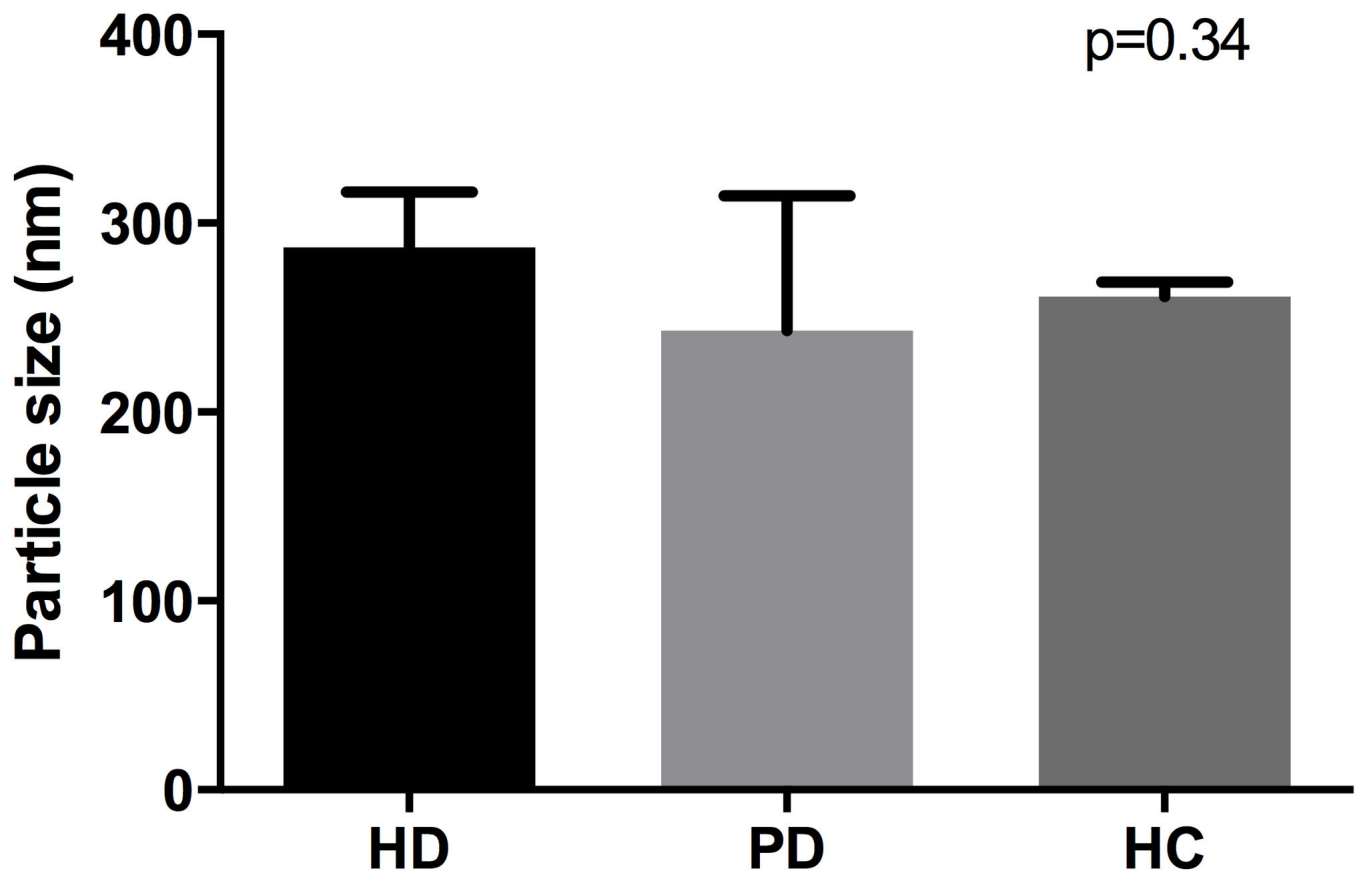
Size range of microvesicles as measured by nanoparticle tracking analysis. Data are shown as the mean±SD for haemodialysis (HD) patients, peritoneal dialysis (PD) patients and healthy controls (HC).

There were no statistically significant differences in the mean sizes of MVs in the HD, PD or control group (287.2±29.4nm, 230.83±71.4nm and 261.3±7.4nm respectively; p=0.34, see Figure 4).

### Circulating MVs in uraemic plasma promote and amplify thrombin generation

**Figure 5:**
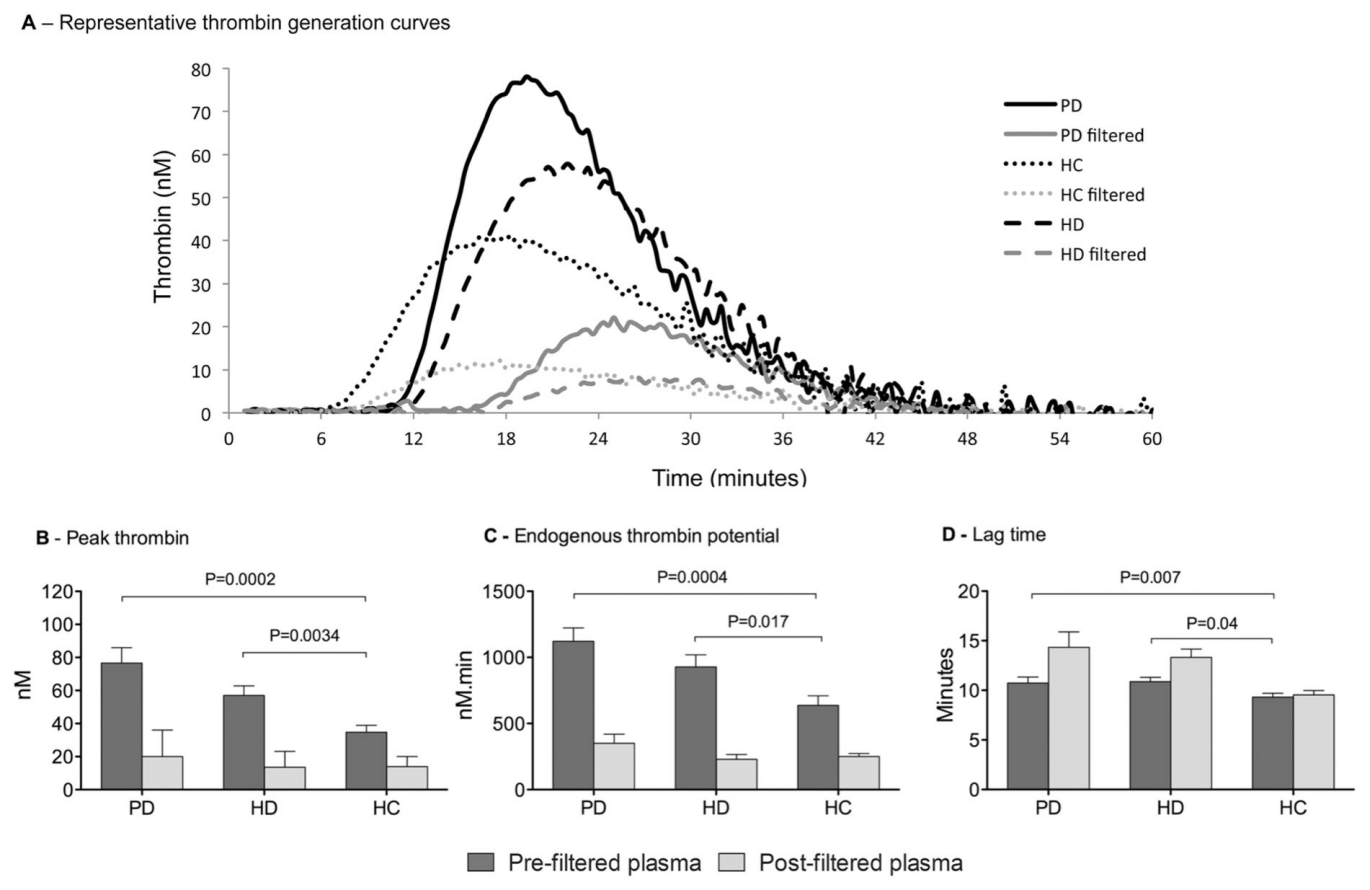
The effects of microvesicles in patients’ plasma on thrombin generation. (A) Representative thrombin generation curves for each subject group (B) Peak thrombin, (in nM.min^-1^) (C) Endogenous thrombin potential (in nM thrombin. min^-1^) and (D) Lag time (in mins), before (dark grey bars) and after (light grey bars) removal of MVs by filtration through a 0.22µM filter. Data are shown as the mean±SD for haemodialysis (HD) patients, peritoneal dialysis (PD) patients and healthy controls (HC).

The presence of MVs in the plasma of patients on dialysis was associated with increased indices of thrombin generation (Figure 5). Representative thrombin generation curves from each of the three subject groups are shown in Figure 5A. PT, which is a sensitive marker for the presence of procoagulant MVs, was higher in both PD and HD patients compared to healthy controls (76.7±38.5nM vs. 34.8±18.1nM, p=0.0002; and 57.1±25.1nM vs. 34.8±18.1nM; p=0.0034 respectively; Figure 5B) but there was no statistically significant difference between the two dialysis groups (p=0.08). ETP was similarly higher in both PD and HD patients compared to healthy controls (1121±421nM.min vs. 637±314nM.min, p=0.0004; and 928±398nM.min vs. 637±314nM.min, p=0.01 respectively; Figure 5C) and again there was no difference observed between dialysis modalities (p=0.17). Both of these parameters are principally indicative of the procoagulant phospholipid component of the MVs. However the LT, was significantly longer in both PD and HD patients compared to controls (10.8±2.5 mins vs. 9.3±1.6 mins; and 10.9±1.8 mins vs. 9.3±1.6 mins, Figure 5D), consistent with reduced levels of coagulation factors previously reported in this patient group [7]. As with the other measures of thrombin generation there was no difference between dialysis groups (p=0.85).

Removal of MVs from plasma by filtration caused a significant reduction in both PT and ETP in all three groups (p<0.0001 for all) and no observed differences remained between the three groups as measured by ANOVA (p=0.15 for PT and p=0.17 for ETP; Figure 5B& C). However, filtration increased the LT in the samples from both PD and HD patients to a similar extent (10.8±2.5 mins vs. 14.3±6.4 mins, p=0.006; and 10.9±1.8 mins vs. 13.3±3.7 mins, p=0.0003) but not in the controls (9.3±1.6 mins vs. 9.4±1.8 mins, p=0.3) (Figure 5D).

**Figure 6:**
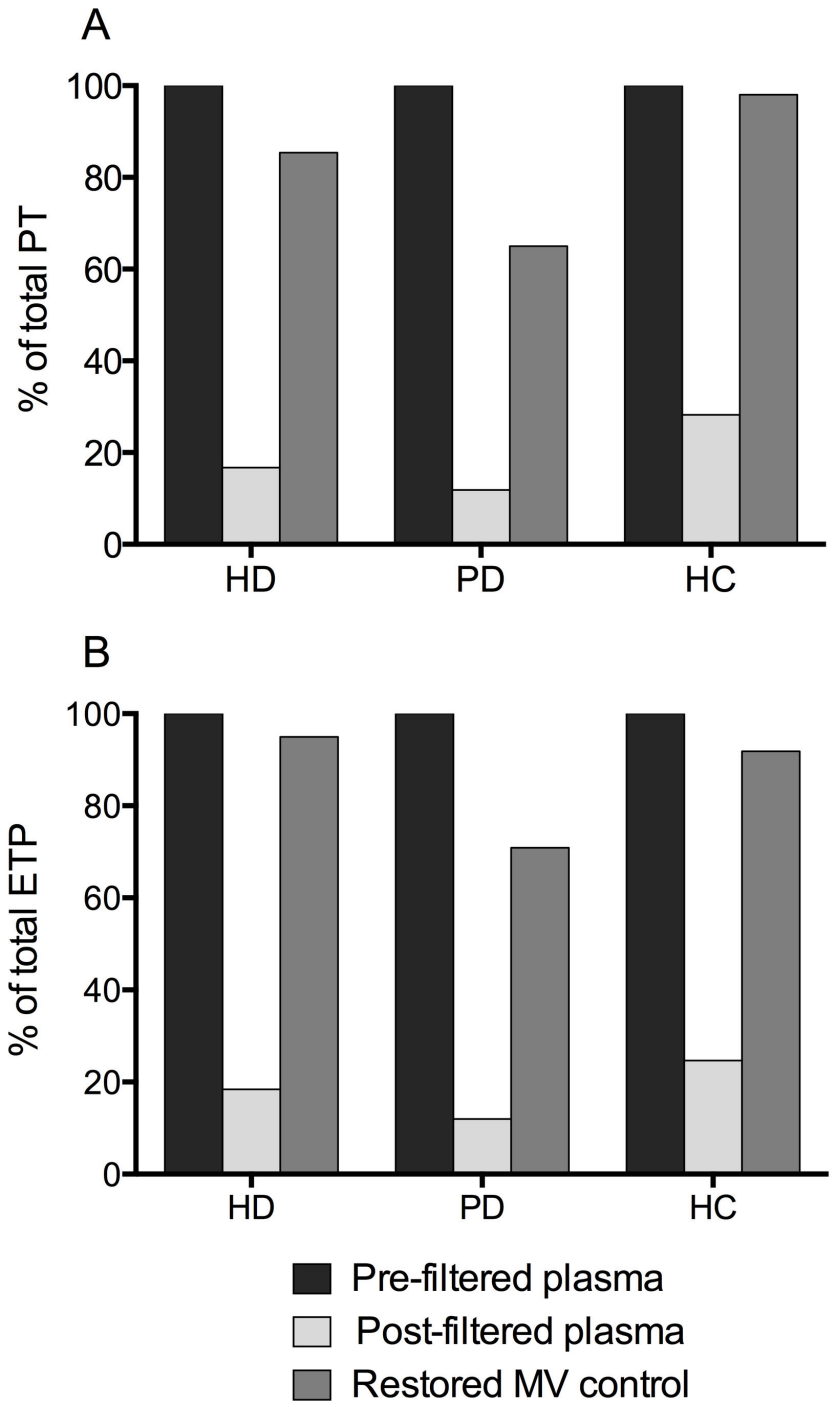
Measurement of thrombin generation in restored microvesicle controls. Peak thrombin (A) and endogenous thrombin potential (B) were measured post-filtration (light grey bars) and in restored MV controls (dark grey bars), and are expressed as a percentage of pre-filtered levels (black bars). HD, haemodialysis; PD, peritoneal dialysis; HC, healthy control.

To confirm that the effects of filtration was predominantly due to the removal of MVs, the re-addition of filtered plasma to prepared MV pellets saw an average restoration of PT and ETP to 82.8% and 85.9% of the level in the unfiltered values respectively. These data are shown for all three subject groups in Figure 6 (n=2 for each group).

### Circulating PMVs and EMVs correlate with thrombin generation in all subject groups

We found robust and statistically significant associations between levels of circulating EMVs and PMVs and both PT and ETP in all subject groups (table 2). There was a statistically significant, inverse correlation between PMVs and LT in both PD and HD patients but not in matched controls. There was also a negative correlation between EMVs levels and LT in each of the subject groups but this only reached statistical significance in the haemodialysis population.

**Table 2:** Correlation between parameters of thrombin generation and microvesicle levels in each subject group.

Group	TG parameter	Mean values	EMV (CD144+ve)	PMV (CD42b+ve)
HD	PT (nM)	57.1±25.1	r=0.52; p=0.02	r=0.7; p=0.002
	ETP (nM)	928±398	r=0.53; p=0.02	r=0.72; p=0.001
	Lag time (mins)	10.9±1.8	r=-0.55; p=0.02	r=-0.44; p=0.08
PD	PT (nM)	76.7±38.5	r=0.62; p=0.02	r=0.56; p=0.03
	ETP (nM)	1121±421	r=0.54; p=0.04	r=0.63; p=0.01
	Lag time (mins)	10.8±2.5	r=-0.38; p=0.18	r=-0.63; p=0.01
HC	PT (nM)	34.8±18.1	r=0.5; p=0.05	r=0.51; p=0.04
	ETP (nM)	637±314	r=0.49; p=0.05	r=0.51; p=0.04
	Lag time (mins)	9.3±1.6	r=-0.23; P=0.4	r=-0.02; p=0.94

PT, peak thrombin measurement; ETP, endogenous thrombin potential; EMV, endothelial derived microvesicles; PMV, platelet derived microvesicles; HD, haemodialysis; PD, peritoneal dialysis; HC, healthy controls. Data are shown, as mean±SD. Correlations were calculated using Pearson’s test.

### Circulating PMVs were significantly lower in patients receiving renin-angiotensin blockade or statins

We compared the relative levels of MVs, determined by Western blotting, between patients taking either statins or an ACE inhibitor / angiotensin-II receptor blocker and those not receiving these agents. Combining both dialysis groups the relative amount of PMVs was significantly lower in those patients on statin therapy (n=23) compared to those not on a statin (n=14) (1.22±0.3 vs. 1.43±0.33, p=0.04). Similarly patients taking either an ACE inhibitor or an angiotensin-II receptor blocker (n=22) had lower levels of EMVs compared to those not on treatment (1.17±0.18 vs. 1.51±0.39, p=0.002). There was a trend towards lower EMVs in those patients on an ACE inhibitor or an angiotensin-II receptor blocker (1.28±0.27 vs. 1.39±0.2, p=0.07) but no difference in EMV levels between patients who were on a statin and those who were not (1.27±0.26 vs. 1.3±0.26, p=0.7).

## Discussion

Our data confirm that circulating plasma levels of MVs (both EMVs and PMVs) are higher in end stage renal patients compared to healthy controls. But, more importantly, we have also shown that these MVs are prothrombotic.

We have combined two novel quantification methods to accurately measure the effects of CKD and dialysis on MV release. Although flow cytometry is still the gold standard of MV measurement even the most sensitive flow cytometers are unreliable in measuring particles of less than 300nm [36]. The data obtained from immunoblotting, which detects specific proteins regardless of their size, has shown that both EMV and PMV subtypes are significantly increased relative to controls and that the relative increase was of a similar order. It is important to appreciate that these values are relative rather than total values and therefore do not conflict with the reported literature that show MV populations are approximately 80% platelet- and 10% endothelial derived [37,38]. Rather, we have shown that dialysis patients have a rise of similar magnitude in MV populations from both cell types compared to their baseline circulating plasma values. In addition, we have shown using the NTA measurements that the total, not just relative, number of circulating MVs of all derivations is significantly increased in our patient populations. Moreover, the average size of particles counted is below that which can be reliably measured using flow cytometry.

We found no difference in levels of either PMVs or EMVs between dialysis modalities. The samples were collected prior to dialysis so we were not exploring the acute effects of dialysis per se, but these results indicate that HD patients do not have inherently (or persistently) greater levels of circulating MVs than PD patients. Although it is thought that the HD treatment itself may be among the stimuli that can affect MV formation [29,39], the exact interplay between HD and levels of circulating MVs still needs further investigation. However, the current data suggests that the elevated levels of circulating MVs observed in dialysis patients are a consequence of the uraemic condition rather than being related to the modality of dialysis used for treatment.

Uraemic toxins such as indoxyl sulphate and *p*-cresol have been shown to increase the release of EMVs from cultured endothelial cells *in vitro* [39], although other uraemic factors may contribute to endothelial activation and subsequent MV generation *in vivo*. Circulating endotoxin is associated with atherosclerosis and cardiovascular risk, and in the HD population has been linked with cardiac injury and reduced survival [40]. Elevated levels of advanced-glycation end products (AGE) are also correlated with poor cardiovascular outcomes in dialysis patients and are strongly linked to the total glucose exposure in patients on PD [41]. Both of these uraemic toxins have the potential to initiate MV release into the circulation [42,43] and demonstrate that although the exact balance of uraemic toxins involved in the process of MV generation between the two dialysis modalities remains unknown, the levels of MVs seen between patient groups are similar.

Regardless of the mechanism of their release, once in the circulation the current data show that MVs may have considerable haemostatic consequences, and that the higher the relative concentration of MVs the greater the influence on thrombin generation. Thrombin generation in the pre-filtered plasma of both HD and PD patients occurred faster, and gave both a higher peak burst and generated a higher total amount of thrombin. This observation is congruent with evidence from other studies that demonstrate comparable degrees of hypercoagulability between HD and PD, suggesting that renal failure *per se* rather than HD is the cause of increased procoagulant activity [44].

Our data is also in general agreement with a recent study by *Trappenburg et. al*. that also showed increased numbers of MVs in patients with end stage renal disease, and increased procoagulant activity in the plasma [45]. There are differences between their study and ours in that they reported only data for ETP measurement, and the assay they used lacked the addition of low level of exogenous TF, making it particularly sensitive to the endogenous level of TF in the samples. Whilst these differences make direct comparison difficult it is of interest that their study demonstrated a lower level of thrombin generation per MV in the patients’ samples compared to the controls, suggesting that the increased MVs generated in the patients had less inherent procoagulant activity that those found in normal plasma.

The thrombin generation assay used in the present study, in which the reaction was primed with a low level (1µM) of TF, is more sensitive to the procoagulant effect of the negatively charged PL on the MVs surface. Both PMVs and EMVs were capable of TF-dependent thrombin generation *in vitro* and displayed elevated levels of procoagulant phospholipid activity, attributed to an increased exposure of phosphatidylserine (PS) on their outer surface. PMVs are reported to display more surface PS than activated platelets [17], but are thought to lack endogenous tissue factor [46]. This is in keeping with our results that showed higher levels of PT and ETP in the plasma of all our subjects. Filtration to remove the MVs resulted in a significant reduction in the PT and ETP in all the groups such that the thrombogenic potential of the filtered plasma of patients on dialysis was not significantly different from that of the control group. This observation, together with the restoration of thrombin generation following reconstitution of endogenous MVs into the filtered plasma, implies that the increased levels of thrombin generation in the patient groups was due to the presence of MVs and not to a soluble factor either in the uraemic plasma or removed as part of the filtration process.

In both dialysis groups, the LT for thrombin generation, which gives an indication of TF activity, was significantly shorter in the presence of MVs in the pre-filtered plasma, whereas the lag time in control subjects was unaffected by the removal of MVs after filtration. This would suggest that MVs in the patients carried more TF than those of controls. However in the pre-filtered samples the patients’ plasma exhibited reduced TF activity, evidenced by the longer lag time compared to controls, which became even more pronounced after filtration to remove the microvesicles (Figure 4D). While at first sight this might seem contradictory to a higher procoagulant activity of the MVs we propose that this may reflect one or more factors that could affect the lag time such as: tissue factor pathway inhibitor (TFPI), the primary inhibitor of the TF-FVIIa complex that initiates the extrinsic coagulation pathway [47]; the presence of uraemic toxins that are not present in the control samples or; that patients have lower levels of other coagulation factors.

The recognition of MVs as prothrombotic, biologically active messengers opens the door to potentially novel therapeutic interventions. So far, several therapies already known to be beneficial in cardiovascular disorders have been reported to reduce circulating MV levels of platelet, monocyte and endothelial cell origin, including calcium channel blockers, statins and angiotensin receptor blockers [23,27]. Our univariate analysis revealed an association between lower levels of PMVs in those patients on statins and renin-angiotensin blockers. We accept that this study was neither designed nor powered to investigate these associations but believe it is still noteworthy as it strengthens the argument that MVs are a potentially modifiable pathogen involved in cardiovascular morbidity and mortality and therefore worthy of future randomised controlled trials.

This study has a number of limitations. It is of cross-sectional design and observational in nature without any prospective data on actual haemostatic consequences or cardiovascular events. We used two independent methods to quantify MVs but with relatively small numbers of subjects studied. Despite this, our study is similar in size to other publications in patients with ESRD and has reproducible results that are entirely in keeping with the current literature. Although accepted methods of plasma preparation were used to optimise MV yield and minimise contamination with platelets, we acknowledge that the samples analysed may also have contained MVs of other subtypes or platelet fragments. However our results show a strong correlation between the two subtypes of MVs measured and thrombin generation. For the matched control group, we relied on self reported medical and medication histories which could have underestimated the prevalence of disease but given that those co-morbidities are known to increase MV concentrations, this would have biased the results towards non-significance.

In conclusion, we have confirmed that PD and HD patients have elevated levels of circulating EMVs and PMVs, which result in significant haemostatic consequences. Whilst the generation of MVs is likely to be multi-factorial in nature, once in the circulation it is these MVs, rather than uraemia *per se*, that are associated with increased thrombin generation and hypercoagulability. MVs may represent a novel and potentially modifiable, non-traditional mediator or predictor of occlusive cardiovascular events in patients with end stage renal disease on dialysis.
